# Reproductive Diseases Are Key Determinants Influencing the Success of Embryo Transfer and Fixed-Time Artificial Insemination in Cattle

**DOI:** 10.3390/ani15172627

**Published:** 2025-09-08

**Authors:** William O. Burgos-Paz, Erly Carrascal-Triana, Sergio Falla-Tapias

**Affiliations:** 1 Centro de Investigación Turipaná, Corporación Colombiana de Investigación Agropecuaria-Agrosavia, Km 13 Vía Montería-Cereté, Córdoba 230550, Colombia; ecarrascal@agrosavia.co; 2 Grupo de Investigación KYRON, Facultad de Medicina Veterinaria y Ciencias Afines, Corporación Universitaria del Huila CORHUILA, Neiva 410010, Colombia; sergio.falla@corhuila.edu.co

**Keywords:** Creole cattle, immune response, prevalence, pregnancy loss, reproductive biotechnologies

## Abstract

Reproductive biotechnologies, such as embryo transfer (ET) and fixed-time artificial insemination (FTAI), are increasingly adopted to enhance genetic progress in tropical cattle production systems. However, the high prevalence of reproductive infectious diseases in tropical regions may compromise reproductive outcomes. This study evaluated the impact of eight reproductive pathogens (*Neospora caninum*, *Leptospira* spp., *Anaplasma* spp., *Babesia* spp., *Trypanosoma* spp., BVDV, IBR, and BLV) on pregnancy success, embryonic loss, and abortion in 296 bovine females subjected to ET and FTAI in Huila, Colombia. Animals were classified into six genetic groups and monitored for pregnancy at 45 and 90 days post-treatment. Logistic regression models were used to evaluate associations between disease prevalence and reproductive outcomes. *Neospora caninum* emerged as the most detrimental pathogen, significantly reducing pregnancy rates (OR = 0.443; *p* = 0.034) and increasing both embryonic loss (OR = 7.35; *p* = 0.073) and abortion risk (ET: OR = 20.3; *p* = 0.0002; FTAI: OR = 3.95; *p* = 0.0436). *Leptospira* spp. and BLV were also associated with increased embryonic losses, whereas *Babesia* spp. and IBR were linked to a reduced risk of embryo resorption because of enhanced care, monitoring, or vaccination. Creole cattle showed lower disease susceptibility than crossbred or commercial breeds. These findings highlight the need for comprehensive disease control, targeted vaccination, and reproductive planning to improve biotechnology outcomes in tropical cattle systems.

## Simple Summary

It is vitally important that scientists are able to describe their work simply and concisely. Reproductive biotechnologies, such as fixed-time artificial insemination (FTAI) and embryo transfer (ET), are increasingly adopted to accelerate genetic progress in cattle production systems. However, infectious diseases can compromise reproductive success, particularly under tropical conditions where surveillance is limited. In this study, we evaluated 296 cows from smallholder dual-purpose herds in Colombia to determine how eight reproductive-related pathogens, age, and genetic group influenced pregnancy outcomes. Almost the entire population was affected by at least one pathogen, and 92.7% of the animals tested positive for two or more diseases. *Neospora caninum* was the most detrimental agent, consistently reducing pregnancy success and increasing losses, while bovine leukosis virus (BLV) and *Leptospira* spp. were also associated with reproductive failure. Creole cattle showed lower susceptibility to infections compared with crossbred and commercial animals, suggesting that local genetic resources contribute resilience under endemic disease pressure. These findings underscore the importance of systematic health surveillance, targeted vaccination, and the integration of local breeds in reproductive planning to improve the success of biotechnologies in tropical cattle systems.

## 1. Introduction

Reproductive efficiency is a key driver of productivity in cattle production systems, particularly in tropical regions where dual-purpose (DP) models—designed for both milk and meat production—are widely implemented [1,2,3]. These systems are highly exposed to multiple environmental and economic stressors [4]. Challenges such as the lack of performance records, poor planning, and limited adoption of best management practices further constrain their productivity [5].

DP systems aim to produce milk in environments where specialized dairy breeds underperform, while simultaneously generating male calves for beef production and females for herd replacement [6]. Although sire selection should prioritize economic efficiency [7], the long-term sustainability of genetic improvement also depends on access to animals with high genetic merit and their adaptability to local conditions [8].

Most DP farms continue to rely on natural mating as their primary reproductive strategy [5]. However, the use of reproductive biotechnologies—such as fixed-time artificial insemination (FTAI) and embryo transfer (ET)—is expanding. These technologies enhance reproductive performance by facilitating the dissemination of genetically evaluated sires/dams and shortening generation intervals [9]. Nevertheless, their effectiveness may be undermined by infectious diseases that compromise fertility and embryonic viability. Notably, pathogens such as *Neospora caninum*, *Leptospira* spp. [10] and hemoparasites [11] are highly prevalent in tropical areas and represent a major constraint.

Tropical environmental and sanitary conditions favor the persistence and transmission of these pathogens, making infectious diseases a persistent challenge to reproductive success [12]. This risk may be greater in DP systems that rely on multiple breed types and their crossbreeds [13], combined with diverse management practices aimed at balancing milk and meat production, thereby increasing susceptibility to disease and reproductive failure. In contrast, single-purpose systems (dairy or beef) usually employ specialized breeds under more uniform management, potentially reducing variability in disease response.

Therefore, the aim of this study was to assess the association between the presence of eight reproductive-relevant infectious diseases and variables such as age and genetic group, in relation to pregnancy outcomes, embryonic loss, and abortion in females subjected to FTAI and ET protocols in Colombia. Understanding these relationships is essential to improving reproductive strategies, promoting genetic progress, and enhancing the overall sustainability of DP cattle systems in tropical environments.

## 2. Materials and Methods

### 2.1. Ethical Approval

This study was conducted following the approval of the Ethics, Bioethics, and Scientific Integrity Committee of the Corporación Colombiana de Investigación Agropecuaria–AGROSAVIA (Act No. 2 of 2021).

### 2.2. Study Area and Population

The study initially used previously reported prevalence data for eight reproductive diseases in 360 bovine females from 150 dual-purpose herds distributed across 24 municipalities within the department of Huila, Colombia [14,15]. Herd selection was conducted in collaboration with livestock associations recognized by institutional authorities; all herds provided certified vaccination records in compliance with national legislation. Consistent with the regional production context, most herds exhibited low technological adoption, comprised fewer than 20 breeding females, and were managed under extensive production systems. The pathogens included protozoal (*Neospora caninum*, *Trypanosoma* spp.), bacterial (*Leptospira* spp., *Anaplasma* spp.), viral (BVDV, IBR, BLV), and hemoparasitic (*Babesia* spp.) agents, all of which are known to compromise reproductive performance in cattle.

All 360 females with available prevalence data underwent a clinical evaluation by a veterinarian specialized in bovine reproduction. Of these, sixty-four clinically healthy animals did not meet the reproductive or body condition requirements established for the biotechnology protocols and were therefore excluded. The final study population thus comprised 296 females, distributed across 125 herds in 24 municipalities. These animals ranged in age from 24 to 140 months and represented the following six predominant genetic groups in the region [6]: Blanco Orejinegro-BON (Creole), Girolando (GYRHOL), F1 Jersey × Holstein (JERHOL), crossbreds with unknown ancestry (MIXED), taurine-type animals (TAURINE), and predominantly zebu-type animals (ZEBUINE).

### 2.3. Embryo Transfer (ET)

Embryos were produced from oocytes obtained from donor females of the Brahman (n = 20), Guzerat (n = 10), and BON (n = 10) breeds, all registered with the national breed association and selected for favorable genetic merit in milk yield and quality. For the male contribution, sexed semen from Holstein bulls (n = 3) and conventional semen from BON bulls (n = 3) were used.

Embryo production was carried out following the protocol [16]. Embryos that reached developmental stage 7 and grade 1 quality, according to the morphological standards established by the International Embryo Transfer Society [17], were previously washed in trypsin and cryopreserved by vitrification and stored in liquid nitrogen until transfer.

Recipient cows were selected based on the protocol [18]. Animals were required to be cyclic, clinically healthy, and free from uterine pathologies and have body condition score between 3.0 and 3.5 (on a 1–5 scale).

Synchronization was carried out using intravaginal progesterone-releasing devices (CIDR or DIB) for 7 to 9 days. On day 6 or 7 of the treatment, prostaglandin F2α (PGF2α) was administered. Upon device removal, ovulation was induced using either estradiol cypionate or estradiol benzoate. Embryo transfer was performed seven days after the detection of synchronized estrus [19].

Pregnancy diagnosis was performed by transrectal ultrasonography (7.5 Mhz linear transducer 75L38EA for Mindray DP-50, Mindray, Shenzhen, China) between days 35 and 45 post-transfer by a single experienced operator and confirmed at 90 days using the same method.

### 2.4. Fixed-Time Artificial Insemination (FTAI)

Females assigned to FTAI were synchronized using the Ovsynch protocol (Calier, Argentina), which consisted of a 100 µg intramuscular injection of GnRH on day 0, followed by a 25 mg intramuscular injection of PGF2α on day 7 and a second 100 µg intramuscular injection of GnRH on day 9. Timed insemination was performed on day 10, using commercial conventional straw semen (0.25 mL, ~20 × 10^6^ spermatozoa) of Holstein (n = 2), BON (n = 6), and Gyr (n = 2) bulls, as well as Hartón del Valle-HDV Creole breed (n = 2) and Guzerat (n = 3) sires, all acquired from recognized commercial suppliers. Pregnancy diagnoses were carried out by transrectal ultrasonography at 45 and 90 days post-insemination.

### 2.5. Statistical Analysis

The association between female age and genetic group with the estimated prevalence of eight reproductive diseases was initially assessed using binary logistic regression models. Additionally, the impact of disease prevalence on reproductive outcomes was evaluated for each reproductive biotechnologies FTAI and ET, based on pregnancy diagnoses performed at 45 and 90 days post-procedure.

Four binary reproductive response variables were defined and analyzed using generalized binary logistic regression models. The models included a set of binary explanatory variables indicating the presence (positive) or absence (negative) of infectious diseases. The linear representation of the model was as follows:

$$log\left(\frac{p}{1-p}\right)=\beta_{0}+\beta_{1}\left({Disease}_{1}\right)+\beta_{2}\left({Disease}_{2}\right)+...+\beta_{k}\left({Disease}_{k}\right)$$

where *p* was the probability of reproductive success, *β*_0_ was the model intercept, *β*_1_, *β*_2_, …, *βₖ* were the estimated coefficients of each disease on the log-odds of reproductive success.

The following reproductive outcomes were used as response variables: (1) confirmed pregnancy at 90 days, (2) initial failure—defined as failure to confirm pregnancy at 45 days, (3) embryonic loss—defined as a change from positive to negative pregnancy status between days 45 and 90, and (4) loss at calving—associated with abortion events. The analyses were conducted separately for animals subjected to FTAI and ET.

Independent variables included the presence/absence of *Neospora caninum*, *Leptospira* spp., *Anaplasma* spp., *Babesia* spp., *Trypanosoma* spp., bovine viral diarrhea virus (BVDV), infectious bovine rhinotracheitis (IBR), and bovine leukemia virus (BLV). Estimated model coefficients (*β_k_*) were exponentiated to obtain odds ratios (ORs) and their corresponding 95% confidence intervals (95% CIs), allowing for interpretation of each disease’s relative impact on reproductive outcomes. Data processing and statistical analyses were conducted using R version 4.5.0 [20].

## 3. Results

A total of 360 female cattle were evaluated to determine their infection status for reproductive-associated diseases [14,15]. Disease prevalence ranged from 40.27% for *Neospora caninum* to 55.55% for IBR. These high prevalence levels underscore the importance of implementing systematic health monitoring, informed animal selection, and reproductive biotechnologies, such as ET and FTAI.

### 3.1. Age and Genetic Group as Determinants of Disease Prevalence

**Figure 1:**
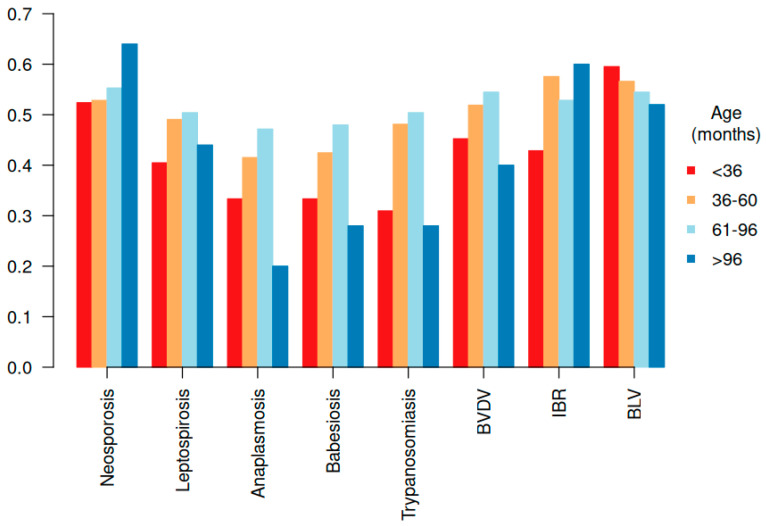
Distribution of the prevalence of reproductive-associated diseases by age group in females.

The effects of age and genetic group on disease prevalence were initially assessed. Age effect showed distinct patterns in disease prevalence. *Neospora caninum* infection was more prevalent in younger animals, whereas the prevalence of hemoparasitic and viral infections increased with age (Figure 1).

Of the 296 females evaluated for reproductive success, only 8 (2.7%) tested negative for all reproductive-related infectious diseases, while 92.6% tested positive for two or more pathogens. For each disease, animals testing negative were used as the reference group. Age did not significantly influence (*p* > 0.05) the prevalence of most of the reproductive diseases evaluated. However, a higher prevalence of Trypanosomiasis was observed in females aged 36–60 months (OR = 2.19; *p* = 0.04) and 61–96 months (OR = 2.30; *p* = 0.03), suggesting an increased risk of infection in older animals compared to younger females. In contrast, the genetic group showed statistically significant associations (*p* < 0.05) with the prevalence of hemoparasitic diseases, such as Anaplasmosis, Babesiosis, and Trypanosomiasis (Table 1).

Compared to the Creole breed (Blanco Orejinegro), MIXED and Taurine females exhibited significantly higher odds ratios (OR) for several reproductive diseases. For instance, JERHOL genetic group demonstrated a notably higher predisposition to Anaplasmosis (OR = 13.70; *p* = 0.01) and Babesiosis (OR = 15.90; *p* = 0.01).

Similarly, the prevalence of Trypanosomiasis was significantly greater across all non-Creole genetic groups (OR > 8, *p* < 0.05), with the highest risk observed in GYRHOL females (OR = 14.90; *p* = 0.01). In the case of viral infections such as BVDV, marginal associations were found in some genetic groups, particularly JERHOL (OR = 6.22; *p* = 0.02), suggesting potential immunological susceptibility. Conversely, no statistically significant associations (*p* > 0.05) were observed for IBR or BLV with respect to age or genetic group.

### 3.2. Influence of Disease Prevalence on Reproductive Success via ET and FTAI

**Figure 2:**
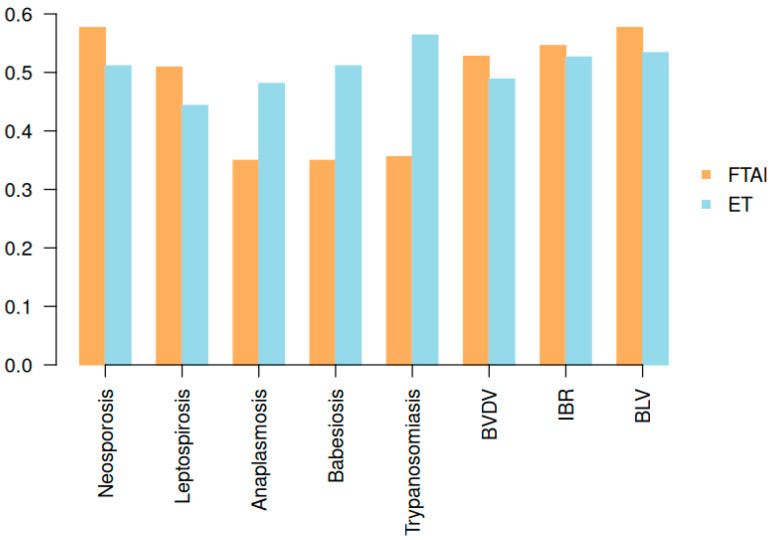
Prevalence of eight reproductive-related infectious diseases in cows managed with fixed-time artificial insemination (FTAI) and embryo transfer (ET).

Figure 2 presents the differences in prevalence of reproductive-related infectious diseases among cows subjected to embryo transfer (ET, n = 133) and fixed-time artificial insemination (FTAI, n = 163).

No statistically significant differences (*p* > 0.05) in the prevalence of *Neospora caninum*, *Leptospira* spp., BVDV, IBR, or BLV were detected between the two reproductive strategies. However, significantly higher prevalence was observed for *Anaplasma* spp. (*p* = 0.03), *Babesia* spp. (*p* = 0.007), and *Trypanosoma* spp. (*p* < 0.001) in animals that underwent ET. Pregnancy diagnoses were conducted at 45 and 90 days post-intervention.

Among the eight females that tested negative for all diseases, pregnancy was successfully established in all using both TE and FTAI protocols. However, only seven calves were ultimately born, as one cow was removed from the herd due to an accident during late gestation.

To further evaluate the potential influence of age and genetic group on reproductive success, these factors were analyzed across the different reproductive phenotypes. Genetic group had a significant effect (*p* < 0.05) only in animals subjected to ET, where Taurine females exhibited a markedly reduced likelihood of confirmed pregnancy compared with Creole females (OR = 0.00384; *p* = 0.000643). In contrast, under the FTAI protocol, age significantly influenced pregnancy success (*p* < 0.05). Cows between 61 and 96 months of age had substantially lower odds of confirmed pregnancy (OR = 0.0502; *p* = 0.0000435) and higher odds of early failure (OR = 0.0405; *p* = 0.0000116) compared with the younger reference group. No significant associations (*p* > 0.05) of age or genetic group were detected for embryonic or calving loss.

Of the 133 cows subjected to ET, 59.39% (n = 79) were diagnosed as pregnant at 45 days, and 50.37% (n = 67) remained pregnant at 90 days. The remaining 66 cows failed to establish or maintain pregnancy. The association between each infectious disease and the four reproductive outcomes (confirmed pregnancy, early failure, embryonic loss, and calving loss) is summarized in Table 2.

Regarding confirmed pregnancy, *Neospora caninum* seropositivity was significantly associated with reduced odds of pregnancy (OR = 0.44; 95% CI: 0.21–0.94; *p* = 0.03). IBR showed a non-significant positive trend (OR = 1.99; *p* = 0.09), while the remaining infections showed no significant (*p*>0.05) associations. For early failure, none of the infections showed statistically significant associations, although *Neospora caninum* (OR = 0.54; *p* = 0.19) and BVDV (OR = 0.56; *p* = 0.13) showed non-significant trends toward reduced odds of early pregnancy success.

In terms of embryonic loss, the logistic regression model could not estimate the effect of *Anaplasma* spp. due to the absence of embryonic losses among seropositive cows (X^2^ = 4.71; *p* = 0.03). Consequently, this variable was excluded from the model for this outcome. Three pathogens showed significant associations with embryonic loss. *Babesia* spp. seropositivity was significantly associated with a lower risk of embryonic resorption (OR = 0.015; 95% CI: 0.0006–0.38; *p* = 0.01), similar to IBR (OR = 0.02; 95% CI: 0.001–0.63; *p* = 0.02). In contrast, BLV infection was significantly associated with a higher risk of embryonic loss (OR = 9.77; 95% CI: 1.05–90.90; *p* = 0.04). Additionally, *Neospora caninum* demonstrated a marginally significant association with increased risk of embryonic resorption (OR = 7.35; *p* = 0.07).

Finally, the presence of *Neospora caninum* was associated with a substantially increased risk of pregnancy loss during gestation (OR = 20.30; *p* = 0.0002). These findings underscore the severe reproductive consequences of this pathogen and its potential to compromise the efficiency of female selection programs in cattle.

In the context of FTAI, notable differences were observed in the impact of infectious disease seropositivity across the four reproductive outcomes (Table 3).

Among the 163 cows evaluated under the FTAI protocol, 41.71% (n = 68) were diagnosed as pregnant at 45 days post-insemination, and 35.58% (n = 58) remained pregnant at the 90-day evaluation. The remaining 105 animals did not achieve pregnancy.

For pregnancy confirmation, none of the evaluated infectious diseases exhibited statistically significant associations (*p* > 0.05). However, *Leptospira* spp. demonstrated a non-significant trend toward reduced pregnancy likelihood (OR = 0.55; 95% CI: 0.28–1.07; *p* = 0.08). Regarding early failure, most diseases were not significantly associated. Notably, IBR infection was significantly associated with reduced pregnancy success (OR = 0.50; 95% CI: 0.26–0.95; *p* = 0.03), indicating a 49.6% lower probability of pregnancy in IBR-positive females. Although *Trypanosoma* spp. infection was not statistically significant (*p* > 0.05), the observed odds ratio suggests that a larger sample size could clarify this potential association.

Concerning embryonic loss, *Leptospira* spp. was the only pathogen significantly associated with an increased risk (OR = 7.91; 95% CI: 1.31–47.80; *p* = 0.02). Finally, similarly to what was observed in the embryo transfer (ET) group, positivity to *Neospora caninum* significantly increased the likelihood of abortion (OR = 3.95; 95% CI: 1.04–15.00; *p* = 0.04).

## 4. Discussion

### 4.1. Differential Economic Impact of Reproductive Biotechnologies

The implementation of reproductive biotechnologies, such as embryo transfer (ET) and fixed-time artificial insemination (FTAI), has played a transformative role in cattle production systems. These technologies have expanded access to superior genetic resources that enhance both productivity and adaptation to local environments. However, their effectiveness is influenced by a variety of factors that introduce substantial variability in outcomes [21,22]. Key limiting factors include gamete quality, environmental stressors, and the health status of animals [23,24]. This variability is particularly pronounced in tropical systems, where heat stress and a high prevalence of infectious diseases can severely hinder the expression of genetic potential [21].

Reproductive failures incur significantly higher economic losses in ET programs compared to FTAI. This is primarily due to the higher operational costs associated with ET, which include embryo production, synchronization protocols, and recipient management. In Colombia, costs related to the recipient and synchronization processes can account for up to 75% of the total cost of a transferred and confirmed embryo at 90 days. As a result, a failed pregnancy in an ET program may lead to financial losses that are 2.8 to 3.5 times greater than those observed in FTAI [25].

Given these cost differences, accurate selection of recipient females is crucial. The variability in reproductive success among females translates directly into differences in economic return [26]. Assessing the infectious disease status of potential recipients is especially important, as it helps reduce gestational losses and improve the economic sustainability of biotechnological interventions [27]. By integrating health status into the selection criteria, producers can optimize pregnancy outcomes and reduce the risk of economic inefficiency in reproductive programs.

### 4.2. Effect of Disease Prevalence on Reproductive Success with ET and FTAI

Health status in Colombian dual-purpose herds is strongly influenced by environmental conditions and management practices [14], and addressing these challenges requires the implementation of effective surveillance and control strategies. In the present study, only 2.7% of females tested negative for all reproductive-related infectious diseases, indicating that nearly the entire population was affected by at least one pathogen. This finding highlights a particularly high burden of multiple infections within the herds, underscoring the relevance of disease prevalence when evaluating reproductive performance.

The implementation of disease-preventive strategies based on risk profiles by age and genetic background may improve reproductive outcomes in systems that use biotechnologies, such as FTAI and ET. Among the diseases evaluated, most showed no significant association with age, except for Trypanosomiasis, which exhibited higher prevalence in middle-aged and older females. Although the immune system typically matures and strengthens with age—potentially mitigating disease impacts on productivity [28,29]—the progressive nature of Trypanosomiasis can lead to anemia, weakness, and subsequent reproductive dysfunction [30]. These findings highlight the importance of age-stratified health monitoring.

For *Neospora caninum*, differences in prevalence between young and adult animals may arise due to both vertical and horizontal transmission pathways [31]. Previous studies [27] have identified access by dogs to water and feed supplies as significant risk factors for horizontal transmission. Nevertheless, the observed prevalence across all age groups suggests that both transmission pathways are active within the population. This underscores the need for rigorous surveillance programs that consider the disease’s epidemiology and associated management practices [32] including dog control and sanitation [33,34]. Increased susceptibility to IBR in adult animals was also observed, consistent with cumulative pathogen exposure and age-related differences in immune response [35].

From a genetic perspective, Creole breeds exhibited lower prevalence of endemic diseases, particularly hemoparasites, compared to zebu or crossbred animals. This supports the hypothesis that local breeds possess enhanced resistance to endemic pathogens due to their genetic adaptation, immunological robustness, and environmental resilience [36,37].

According to the FAO’s Domestic Animal Diversity Information System (DAD-IS), as of 2010, at least 17 cattle breeds worldwide have been identified as resistant to Trypanosomiasis, 2 to Anaplasmosis, 4 to Babesiosis, and 9 to bovine leukosis, among others [38]. The present results provide evidence from a South American context, suggesting that Colombian Creole breeds represent a valuable genetic resource for coping with endemic diseases. This resistance could be strategically integrated into breeding programs aimed at resilience under climate change and health threats.

Regarding the impact of diseases on reproductive outcomes, the magnitude and direction of pathogen effects varied between reproductive biotechnologies. For example, *Neospora caninum* significantly reduced pregnancy rates and increased embryonic loss in ET recipients, while its impact in FTAI protocols was less pronounced and not statistically significant. For this study, all transferred embryos were washed in trypsin according to International Embryo Technology Society (IETS) standards. Previous studies have demonstrated that ET procedures, even using embryos from seropositive donors, do not transmit Neosporosis [39,40,41].

Bovine herpesvirus 1 (BoHV-1), the causative agent of IBR, establishes latent infections, making infected animals lifelong carriers and potential sources of viral reactivation [42]. In this study, IBR was associated with increased embryonic loss in ET protocols, likely due to reactivation during critical stages of pregnancy. Conversely, in FTAI, IBR infection was more closely linked to early pregnancy failure, possibly due to immune dysregulation at conception.

Leptospirosis was significantly associated with embryonic loss in FTAI protocols but not in ET. While disease prevalence was not significantly higher in the FTAI group (*p* > 0.05), the presence of *Leptospira* spp. in the uterus can increase interleukin-6 (IL-6) and trigger a pro-inflammatory environment that compromises embryo survival [43]. The pathogen invades the placenta between days 14 and 60 of gestation and is also associated with infertility due to uterine and ovarian infection, extended calving intervals, and delayed conception—factors that translate into economic losses [44]. Therefore, screening the health status of semen used in FTAI is critical, as semen from infected bulls can directly transmit the pathogen.

A noteworthy finding was the strong association between BLV and embryonic loss in ET programs (OR = 9.77, *p* < 0.05). This effect was not observed in FTAI, warranting further investigation given previous reports linking BLV to abortion risk [45].

Specific analysis of post-implantation fetal losses (abortions) confirmed the relevance of *Neospora caninum*, with highly significant effects in both FTAI (OR = 3.95, 95% CI: 1.04–15.00) and ET (OR = 20.30, 95% CI: 4.11–100), the latter showing a more pronounced effect. Previous studies have confirmed the abortive role of *Neospora caninum* in cattle, especially between 3 and 7 months of gestation due to interactions between the parasite, fetal immune development, and maternal immune response [46,47]. The stronger effect observed in ET suggests that latent infections may be more detrimental when embryos are introduced externally, likely due to uterine inflammation compromising implantation and embryo survival [48].

Interestingly, *Babesia* spp. was associated with a protective effect against embryonic loss (OR = 0.01, *p* = 0.01). Although unexpected—given its known effects on anemia and reproductive suppression—this may reflect enhanced animal care and monitoring in chronically infected individuals, which could improve pregnancy maintenance [14]. Similarly, while IBR is a known cause of abortion in acute infections, prior immunity (e.g., via vaccination) may reduce viral activity during gestation, thus lowering abortion risk [49].

The observed differences in disease effects across biotechnologies may be attributed to physiological and immunological responses specific to each protocol. ET involves direct manipulation of the reproductive tract and stimulates anti-inflammatory responses to support embryo retention [50]. However, this immune modulation could facilitate reactivation of latent infections such as *Neospora caninum*, potentially triggering uterine inflammation and compromising embryonic development [51].

The disaggregated analysis of reproductive outcomes (confirmed pregnancy, early failure, embryonic loss, and calving loss) proved valuable in identifying critical stages at which specific pathogens exert their effects. Overall, the evaluation of reproductive outcomes revealed that *Neospora caninum* had the most consistent negative impact, being associated with both a reduced probability of pregnancy and increased abortion rates across reproductive technologies (ET and FTAI). This detailed understanding can inform the design of more effective disease prevention and control strategies tailored to each reproductive biotechnology.

## 5. Conclusions

This study shows that Blanco Orejinegro (BON) Creole breed females exhibit lower susceptibility to hemoparasitic and viral infections than crossbred and commercial cattle. Among the pathogens assessed, *Neospora caninum* emerged as the infectious agent most consistently and negatively associated with reproductive success, while BLV and *Leptospira* spp. also contributed to embryonic losses. These findings underscore the importance of comprehensive control programs—including vaccination, health monitoring, and age-specific management—to improve reproductive efficiency and the performance of ET and FTAI. Moreover, the monitoring of zoonotic agents such as *Leptospira* spp. should be prioritized under a One Health framework.

## Figures and Tables

**Table 1:** Genetic group and age of females: effects on the prevalence of reproductive-associated diseases.

	Level *	OR	IC_inf	IC_sup	*p* Value
Neosporosis	Age 36–60	0.99	0.48	2.05	0.98
	Age 61–96	1.06	0.52	2.15	0.89
	Age > 96	1.60	0.56	4.55	0.38
	GYRHOL	0.83	0.23	2.93	0.77
	JERHOL	2.10	0.49	8.94	0.32
	MIXED	0.70	0.21	2.27	0.55
	Taurine	0.73	0.21	2.57	0.62
	Zebuine	0.50	0.13	1.89	0.30
Leptospirosis	Age 36–60	1.41	0.68	2.94	0.36
	Age 61–96	1.51	0.74	3.08	0.260
	Age > 96	1.12	0.40	3.12	0.82
	GYRHOL	1.92	0.51	7.17	0.33
	JERHOL	1.89	0.46	7.78	0.38
	MIXED	2.03	0.59	6.96	0.26
	Taurine	2.52	0.68	9.38	0.17
	Zebuine	2.87	0.71	11.60	0.14
Anaplasmosis	Age 36–60	1.42	0.66	3.05	0.36
	Age 61–96	1.74	0.83	3.65	0.14
	Age > 96	0.50	0.15	1.66	0.26
	GYRHOL	7.56	0.90	63.80	0.06
	JERHOL	13.70	1.53	123.00	0.01
	MIXED	8.06	1.01	64.40	0.04
	Taurine	10.30	1.22	86.40	0.03
	Zebuine	6.99	0.78	62.70	0.08
Babesiosis	Age 36–60	1.48	0.70	3.17	0.31
	Age 61–96	1.80	0.85	3.77	0.12
	Age > 96	0.772	0.25	2.35	0.65
	GYRHOL	9.16	1.09	76.90	0.04
	JERHOL	15.90	1.78	143.00	0.01
	MIXED	7.96	1.00	63.40	0.05
	Taurine	11.30	1.34	94.10	0.02
	Zebuine	7.92	0.90	70.30	0.06
Trypanosomiasis	Age 36–60	2.19	1.01	4.75	0.04
	Age 61–96	2.30	1.08	4.90	0.03
	Age > 96	0.88	0.29	2.69	0.82
	GYRHOL	14.90	1.77	126.00	0.01
	JERHOL	14.10	1.57	127.00	0.02
	MIXED	8.54	1.07	68.20	0.04
	Taurine	12.40	1.48	104.00	0.02
	Zebuine	11.20	1.26	99.10	0.03
BVDV	Age 36–60	1.39	0.67	2.88	0.38
	Age 61–96	1.45	0.71	2.95	0.31
	Age > 96	0.83	0.30	2.34	0.73
	GYRHOL	3.31	0.80	13.70	0.10
	JERHOL	6.22	1.35	28.70	0.02
	MIXED	3.75	0.98	14.4	0.05
	Taurine	2.62	0.64	10.8	0.18
	Zebuine	3.79	0.85	16.8	0.08
IBR	Age 36–60	1.87	0.90	3.89	0.10
	Age 61–96	1.47	0.72	3.01	0.29
	Age > 96	2.28	0.81	6.45	0.12
	GYRHOL	2.56	0.72	9.13	0.15
	JERHOL	2.73	0.69	10.80	0.15
	MIXED	2.05	0.63	6.69	0.23
	Taurine	2.25	0.63	7.98	0.21
	Zebuine	1.16	0.30	4.47	0.83
BLV	Age 36–60	0.91	0.44	1.91	0.819
	Age 61–96	0.85	0.41	1.74	0.65
	Age > 96	0.81	0.29	2.23	0.68
	GYRHOL	1.62	0.47	5.59	0.45
	JERHOL	0.74	0.19	2.84	0.66
	MIXED	1.59	0.50	5.01	0.43
	Taurine	1.71	0.50	5.90	0.40
	Zebuine	1.44	0.39	5.36	0.59

* Reference groups: age < 36 months and genetic group Creole.

**Table 2:** Effect of infectious disease positivity on four phases of reproductive success in cows subjected to embryo transfer (ET).

Infectious Disease	Confirmed Pregnancy OR (IC 95%)	Early Failure OR (IC 95%)	Embryonic Loss OR (IC 95%)	Calving Loss OR (IC 95%)
Neosporosis	0.44 (0.21–0.94) *	0.54 (0.25–1.17)	7.35 (0.83–65.10) ^+^	20.30 (4.11–100.00) **
Leptospirosis	0.88 (0.41–1.87)	0.73 (0.35–1.56)	0.35 (0.03–3.74)	1.27 (0.28–5.72)
Anaplasmosis	1.13 (0.30–4.35)	0.68 (0.17–2.71)	—	1.10 (0.10–11.60)
Babesiosis	1.52 (0.40–5.80)	1.13 (0.29–4.44)	0.01 (0.0006–0.38) *	0.65 (0.04–11.10)
Trypanosomiasis	0.66 (0.19–2.36)	0.98 (0.28–3.49)	2.41 (0.18–31.60)	0.41 (0.04–3.99)
BVDV	0.84 (0.40–1.77)	0.56 (0.26–1.19)	0.08 (0.006–1.27) ^+^	1.41 (0.34–5.87)
IBR	1.99 (0.89–4.45) ^+^	1.23 (0.55–2.77)	0.02 (0.001–0.63) *	0.99 (0.21–4.70)
BLV	0.61 (0.30–1.28)	0.68 (0.33–1.42)	9.77 (1.05–90.90) *	1.22 (0.29–5.12)

** *p* < 0.01, * *p* < 0.05, ^+^ *p* < 0.10.

**Table 3:** Effect of seropositivity to infectious agents on four reproductive outcomes in cattle subjected to fixed-time artificial insemination (FTAI).

Infectious Disease	Confirmed Pregnancy OR (IC 95%)	Early Failure OR (IC 95%)	Embryonic Loss OR (IC 95%)	Calving Loss OR (IC 95%)
Neosporosis	0.87 (0.44–1.70)	1.03 (0.54–1.99)	1.87 (0.29–12.20)	3.95 (1.04–15.00) *
Leptospirosis	0.55 (0.28–1.07) ^+^	0.83 (0.44–1.57)	7.91 (1.31–47.80) *	1.47 (0.42–5.11)
Anaplasmosis	0.57 (0.15–2.18)	0.69 (0.20–2.45)	1.92 (0.16–23.00)	2.68 (0.42–17.00)
Babesiosis	1.75 (0.56–5.44)	1.26 (0.43–3.72)	0.38 (0.03–4.94)	0.58 (0.08–3.99)
Trypanosomiasis	1.37 (0.46–4.03)	1.33 (0.47–3.73)	0.94 (0.14–6.41)	1.86 (0.40–8.74)
BVDV	1.60 (0.83–3.10)	1.74 (0.92–3.30) ^+^	0.67 (0.13–3.49)	1.14 (0.32–4.09)
IBR	0.66 (0.34–1.29)	0.50 (0.26–0.95) *	0.22 (0.03–1.51) ^+^	0.37 (0.10–1.41)
BLV	0.87 (0.44–1.73)	0.98 (0.50–1.89)	2.14 (0.39–11.80)	0.77 (0.22–2.65)

* *p* < 0.05, ^+^ *p* < 0.10.

## Data Availability

The data presented in this study are available on request from the corresponding author due to privacy reasons.
